# Parent’s perspectives of the pathway to diagnosis of childhood cancer: a matter of diagnostic triage

**DOI:** 10.1186/s12913-020-05821-2

**Published:** 2020-10-22

**Authors:** Line Hjøllund Pedersen, Ayo Wahlberg, Marie Cordt, Kjeld Schmiegelow, Susanne Oksbjerg Dalton, Hanne Bækgaard Larsen

**Affiliations:** 1 Department of Paediatrics and Adolescent Medicine, Rigshospitalet, Copenhagen, Denmark; 2 Department of Anthropology, University of Copenhagen, Copenhagen, Denmark; 3 Institute of Clinical Medicine, University of Copenhagen, Copenhagen, Denmark; 4 Danish Cancer Society Research Centre, Survivorship and Inequality in Cancer, Danish Cancer Society, Copenhagen, Denmark; 5 Department of Clinical Oncology & Palliative Care, Zealand University Hospital, Naestved, Denmark

**Keywords:** Early diagnosis, Diagnostic pathway, Childhood cancer, Parent, Primary care, Secondary care, Triage, Qualitative research, Theory development, Model

## Abstract

**Background:**

Early diagnosis is crucial for the treatment of childhood cancer as it in some cases can prevent progression of disease and improve prognoses. However, childhood cancer can be difficult to diagnose and barriers to early diagnosis are multifactorial. New knowledge about factors influencing the pathway to diagnosis contribute to a deeper understanding of the mechanisms that influence this time span. Qualitative research in the field is sparse but can be expected to lead to additional useful insights that could contribute to efforts shorten time to diagnosis. The purpose of this study was to explore parents’ experiences of the pathway to diagnosis in the time between their noticing bodily or behavioural changes and their child’s diagnosis.

**Methods:**

The study is a qualitative interview study carried out in large Danish hospital. Thirty-two interviews with a total of 46 parents of children with cancer were included for analysis. The children were diagnosed with haematological cancers (*n* = 17), solid tumours (*n* = 9) or brain tumours (*n* = 6). Data were analysed applying the theoretical model of pathways to treatment and an inductive-deductive approach. A revised ‘diagnostic triage’ model was developed and validated by member checking.

**Results:**

The pathway to diagnosis was influenced by various factors which we present as consistent parts of a new diagnostic triage model. Each factor impacts the level of urgency assigned to bodily and behavioural changes by parents, general practitioners and specialists. The model of diagnostic triage was developed and validated to understand mechanisms influencing time from the point parents notice changes in their child to diagnosis. The model identifies dynamic movement between parental triage in everyday life and professional triage in a healthcare system, both affecting appraisal and case escalation according to: 1) the nature of bodily and behavioural changes, 2) parental intuition, 3) social relations, 4) professional-child-parent interaction, and 5) specialist-child-parent interaction.

**Conclusions:**

Diagnostic triage is a model which explains mechanisms that shape the pathway to diagnosis. It is a contribution aimed at supporting the clinical diagnostic process, that ultimately could ensure more timely testing, referral and diagnosis, and also a novel theoretical framework for future research on diagnostic pathways.

**Supplementary information:**

**Supplementary information** accompanies this paper at 10.1186/s12913-020-05821-2.

## Background

Cancer is among the leading causes of child death in high income countries [1]. Early diagnosis is crucial for the treatment of childhood cancer as it in some cases can prevent progression of disease and improve prognoses [2–6]. However, evidence on the prognostic impact of time to diagnosis (TTD) is mixed and differs among cancer types [2].

The barriers to adequate and timely diagnosis are multifactorial and related to parent, patient, the healthcare system (HCS) and the type of cancer [2, 7–10]. Studies suggest that main factors related to delay are: age at diagnosis, parents’ level of education, type of cancer and presentation of symptoms [2, 8–11]. Misinterpretation of symptoms and undeveloped communication skills among children for conveying their symptoms and illness all contribute to a prolonged TTD [3, 4, 10, 12, 13] adding to the possible progression of the cancer.

There is only limited research available about barriers to early diagnosis of childhood cancer and most of this research uses quantitative methods. Studies on diagnostic delay have examined predictors and medical systems [2, 10, 14–17], showing how differences in HCS may account for variation in TTD [18–21]. Studies of parents’ experiences and accounts of the pathway to diagnosis have also been conducted showing that, given the rarity of childhood cancer, parents do not immediately interpret their children’s symptoms in the context of cancer, and many symptoms are initially managed without consulting a general practitioner (GP) [22]. The decision to consult their GP is influenced by physical, psychological and social factors [8, 9, 23, 24]. Once a GP is consulted, parents seek explanations and are persistent about obtaining a diagnosis [11]. Experienced delay is associated with feelings of guilt including failing to correctly interpret symptoms, consult a GP at an earlier stage or demand further examinations and tests [25].

Clearly, the period prior to diagnosis has a psychological and possibly also a prognostic impact dependent on the type of cancer. For all these reasons, delay remains a crucial challenge to solve in cancer treatment. The term “delay” has frequently been used in the literature with different definitions e.g. “an unqualified period of time between symptom onset and definitive diagnosis” [13]. However, “delay” has been criticized for being value laden, inaccurate [13, 26] and not recognizing that help-seeking occurs within a context of everyday considerations, priorities and activities [27]. In recognizing this critique, we perceive the pathway to diagnosis as a time period where daily events and processes in the home, during GP consultations and at specialist clinics influence the speed of the diagnostic process. It is not our intention to assess whether an individual pathway has been unacceptably long, or whether the children in our study received a timely diagnosis, rather we aim to explore parents’ perspectives on the pathway to their child’s diagnosis.

Qualitative research in the field is sparse but can lead to useful insights that could contribute to the shortening of time to diagnosis. Parents can provide unique insights into appraisal, help-seeking and interactions with the HCS based on their experiences. Their accounts of the pathway to diagnosis can help clarify variations in pathways prior to diagnosis in investigations of barriers for early diagnosis. New knowledge about factors (barriers and triggers) influencing the pathway contribute to a deeper understanding of the mechanisms that influence the time from the detection of first bodily or behavioural change(s) to diagnosis. This, in turn, can help identify targets for possible interventions to improve early detection and diagnosis. The purpose of this study was therefore to explore and analyse parents’ experiences of the pathway to diagnosis in the time between noticing bodily or behavioural changes and their child’s diagnosis.

### New contribution

**Fig. 1:**
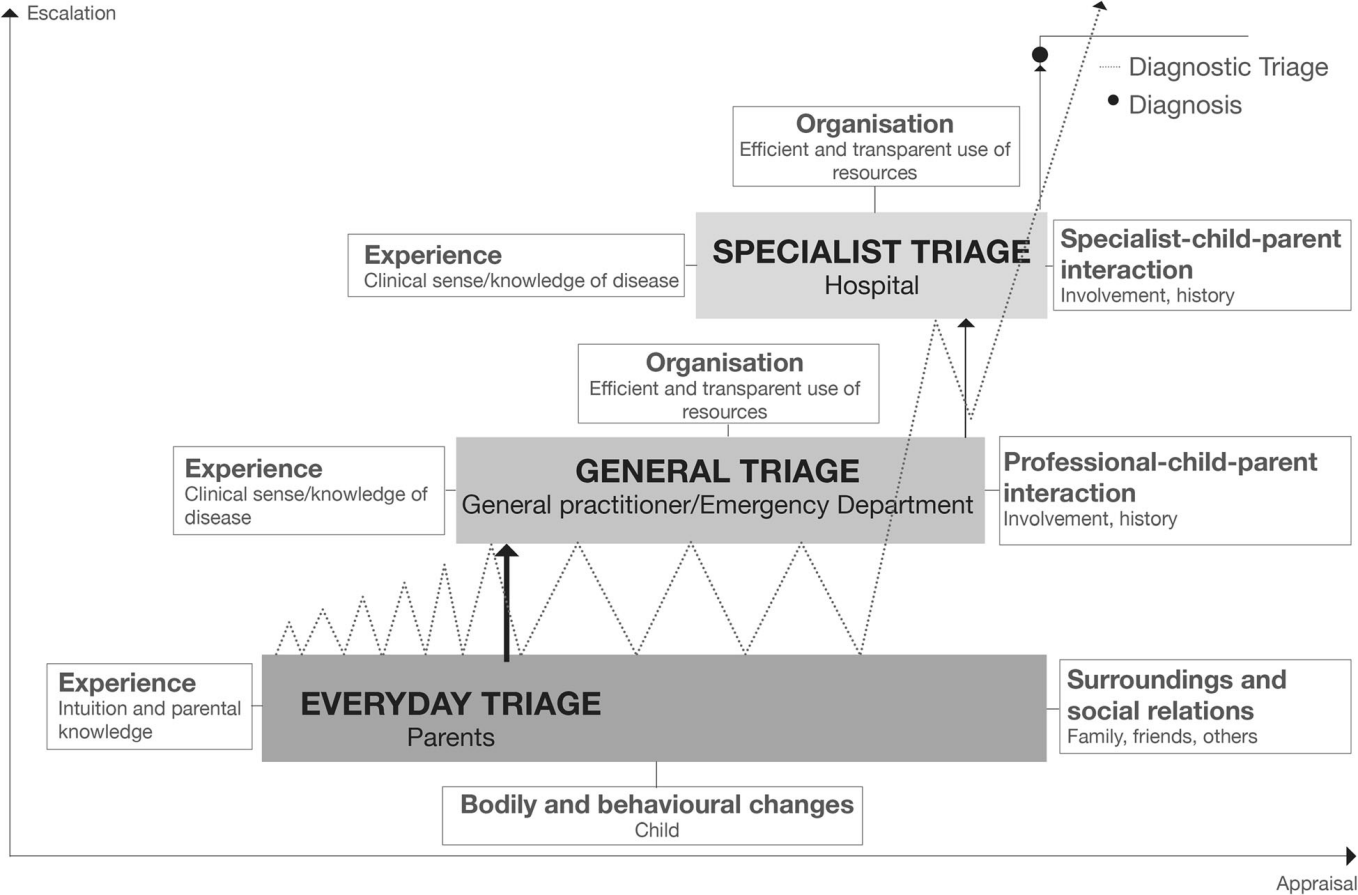
Diagnostic triage: The process of appraisal, help-seeking, negotiation and escalation in the pathway from detection of bodily and behavioural changes to diagnosis. The zig-zag line illustrates the mechanisms of diagnostic triage. It is a continual and dynamic process of appraisal, escalations (peak upwards) and de-escalations (peak downwards) along the pathway to diagnosis covering three different forms of triage. The dynamic movements take place between everyday triage, general triage and specialist triage which are regulated by various factors. The multiple peaks in the zig-zag line visualises how families can move back and forth between the different levels of triage before experiencing progress and obtaining the diagnosis

This paper contributes with new knowledge on two levels. First, it presents factors shaping the pathway to diagnosis from parent’s perspectives analysed using “The model of pathways to treatment” [7]. Secondly, it proposes an explanatory model called ‘diagnostic triage’ (Fig. 1) which explains everyday mechanisms that shape the pathway from the detection of bodily or behavioural change(s) to diagnosis.

## Methods

### Design and setting

The study has a qualitative design with semi-structured interviews and was carried out in accordance with guidelines for reporting of qualitative research [28]. It was conducted in Denmark, a welfare state with free universal health care where the GP serves as a gatekeeper in primary care, responsible for initial assessments and referral to further examinations or specialist consultations.

### Participants and data collection

The study is based on data from 32 interviews with parents of children with cancer (Table 1). The children were diagnosed with haematological cancers (*n* = 17), solid tumours (*n* = 9) or brain tumours (*n* = 6). For each case either one parent (18 interviews) or two parents (14 interviews) took part in the interview corresponding to 46 parents participating in the study.

**Table 1:** Characteristics of 32 included cancer patients and 46 associated interviews of parents

Interview/case #	Diagnosis	Child’s age group at diagnosis (years)	Sex	Time interval: From first appearance of change to diagnosis	Interview person (P)	Parental education	Parent’s age group at interview (years)	Number of children in family	Validation of model as part of interview
1	Haematological cancer	0–4	F	5 months	Mom/P1	Higher	Missing	2	No
2	Brain tumour	10–14	F	8 months	Mom/P2	Higher	Missing	2	No
3	Haematological cancer	5–9	M	4 months	Mom/P3	Higher	> 40	3	No
4	Haematological cancer	10–14	M	4 months	Mom/P4 Dad/P5	Higher Higher	Missing Missing	2	No
5	Solid tumour	10–14	F	9 months	Mom/P6	Higher	> 40	3	No
6	Haematological cancer	0–4	M	2 weeks	Dad/P7	Higher	Missing	1	No
7	Haematological cancer	10–14	M	2.5 weeks	Mom/P8	Higher	> 40	3	No
8	Haematological cancer	0–4	M	1 month	Dad/P9	Higher	31–40	2	No
9	Haematological cancer	0–4	F	4 months	Mom/P10	Higher	31–40	3	No
10	Brain tumour	0–4	M	3 years	Dad/P11	Higher	Missing	2	No
11	Haematological cancer	0–4	M	4 months	Mom/P12	Higher	Missing	1	No
12	Solid tumour	10–14	F	3 weeks	Mom/P13 Dad/P14	Higher Medium	> 40 > 40	2	No
13	Solid tumour	15–18	M	1 month	Mom/P15 Dad/P16	Higher Higher	> 40 > 40	2	No
14	Solid tumour	0–4	M	4 months	Mom/P17 Dad/P18	Higher Higher	31–40 31–40	2	No
15	Haematological cancer	0–4	M	3 months	Mom/P19 Dad/P20	Higher Medium	31–40 31–40	2	No
16	Solid tumour	10–14	F	1.5 months	Mom/P21 Dad/P22	Higher Higher	> 40 > 40	3	No
17	Haematological cancer	5–9	F	5 months	Mom/P23 Dad/P24	Higher Medium	31–40 31–40	2	No
18	Haematological cancer	0–4	F	2 weeks	Mom/P25 Dad/P26	Higher Higher	> 40 > 40	3	No
19	Brain tumour	5–9	M	Missing	Mom/P27 Dad/P28	Higher Medium	> 40 > 40	2	No
20	Haematological cancer	0–4	M	1.5 months	Mom/P29 Dad/P30	Medium Medium	≤30 31–40	3	No
21	Solid tumour	0–4	F	1 week	Mom/P31 Dad/P32	Higher Medium	31–40 > 40	2	No
22	Solid tumour	10–14	M	2 weeks	Mom/P33 Dad/P34	Medium Higher	31–40 31–40	2	No
23	Haematological cancer	15–18	F	1 week	Dad/P35	Higher	Missing	4	No
24	Haematological cancer	10–14	M	1 month	Mom/P36	Higher	Missing	2	No
25	Haematological cancer	0–4	M	11 days	Mom/P37 Dad/P38	Higher Short	Missing Missing	4	No
26	Haematological cancer	10–14	M	1 week	Mom/P39	Higher	> 40	2	No
27	Haematological cancer	10–14	M	3 months	Mom/P40 Dad/P41	Higher Higher	31–40 31–40	2	Yes
28	Solid tumour	10–14	M	2,5 years	Mom/P42	Medium	> 40	3	Yes
29	Brain tumour	0–4	M	11 months	Mom/P43	Higher	31–40	2	Yes
30	Solid tumour	15–18	F	6 months	Mom/P44	Medium	> 40	1	Yes
31	Brain tumour	0–4	M	2 ¼ years	Mom/P45	Medium	31–40	2	Yes
32	Brain tumour	15–18	M	12 months	Mom/P46	Higher	> 40	2	Yes

Time intervals are estimates reported by parents. Parental education is highest achieved degree. Three categories are created for educational level: Short (Primary/secondary school and high school diploma), Medium (Vocational education and Short higher education (≤2 years)), Higher (Bachelor degree (≤4 years), Master’s degree and PhD degree) The categories are based on the International Standard Classification of Education [29]. Stepparents as interview persons are also designated mom/dad

The study was divided into two parts. The first part included 26 interviews with parents recruited through convenience sampling (case 1–26) (Table 1). Parents were approached at a paediatric oncology ward at a large Danish hospital. They were invited to participate and asked to read an information sheet before deciding whether they would participate. After a minimum of 3 days hereafter they were contacted by telephone or email.

In the interviews, parents retrospectively provided accounts of the time from noticing changes in their child to diagnosis, with a specific interest in factors influencing the pathway including uncertainties, obstacles and escalation triggers. An interview guide with primarily open-ended questions encouraged parents to elaborate on experiences with appraisals, help-seeking, medical encounters and eventually obtaining a diagnosis. New themes were added as they emerged during the interviews. The interview guide developed for this study is provided as Additional file 1.

The second part comprised six interviews (case 27–32) with parents recruited through purposive sampling. They were selected to validate the diagnostic triage model (which was developed on data from the first 26 interviews). They were recruited by experienced clinicians (specialists) who deemed the families to have had a noticeably long and/or frustrated pathway to diagnosis. Their experiences and extensive knowledge of barriers and triggers along the way were of great value in the process of validation. The families were contacted on the ward, at the out-patient clinic or by phone. All invited parents agreed to participate.

The six member checking interviews were carried out with either one or two parents participating. The interviews were divided into two steps: Step 1) The parents were asked to share their experiences in a similar way as all other parents using the same interview guide. This was to explore whether new themes would emerge. Step 2) This second step was the validation part. In the same interview, parents were presented with the diagnostic triage model and asked systematically to comment on their experiences in relation to the model. The parents had the opportunity to comment and express their agreement and/or disagreement of whether the model was a truthful and reliable way of perceiving and presenting the pathway to diagnosis (see Additional file 1).

The 32 interviews, which lasted from 30 min to 2 h, were carried out by MC, AW and LHP over a three-year period from 2016 to 2019 in a private hospital room or in the parents’ homes. Participants, who provided informed consent, were informed that the interviews would be anonymised. Interviews were recorded, transcribed verbatim and analysed by four researchers (LHP, AW, MC and HBL). Transcripts were imported to NVivo12. Parents are referred to by letter (P).

### Analysis

Data analyses were conducted in a number of steps: 1) Reading the interviews, 2) Coding, 3) Summary of each patient, 4) Development of model, 5) Member checking.

**Fig. 2:**
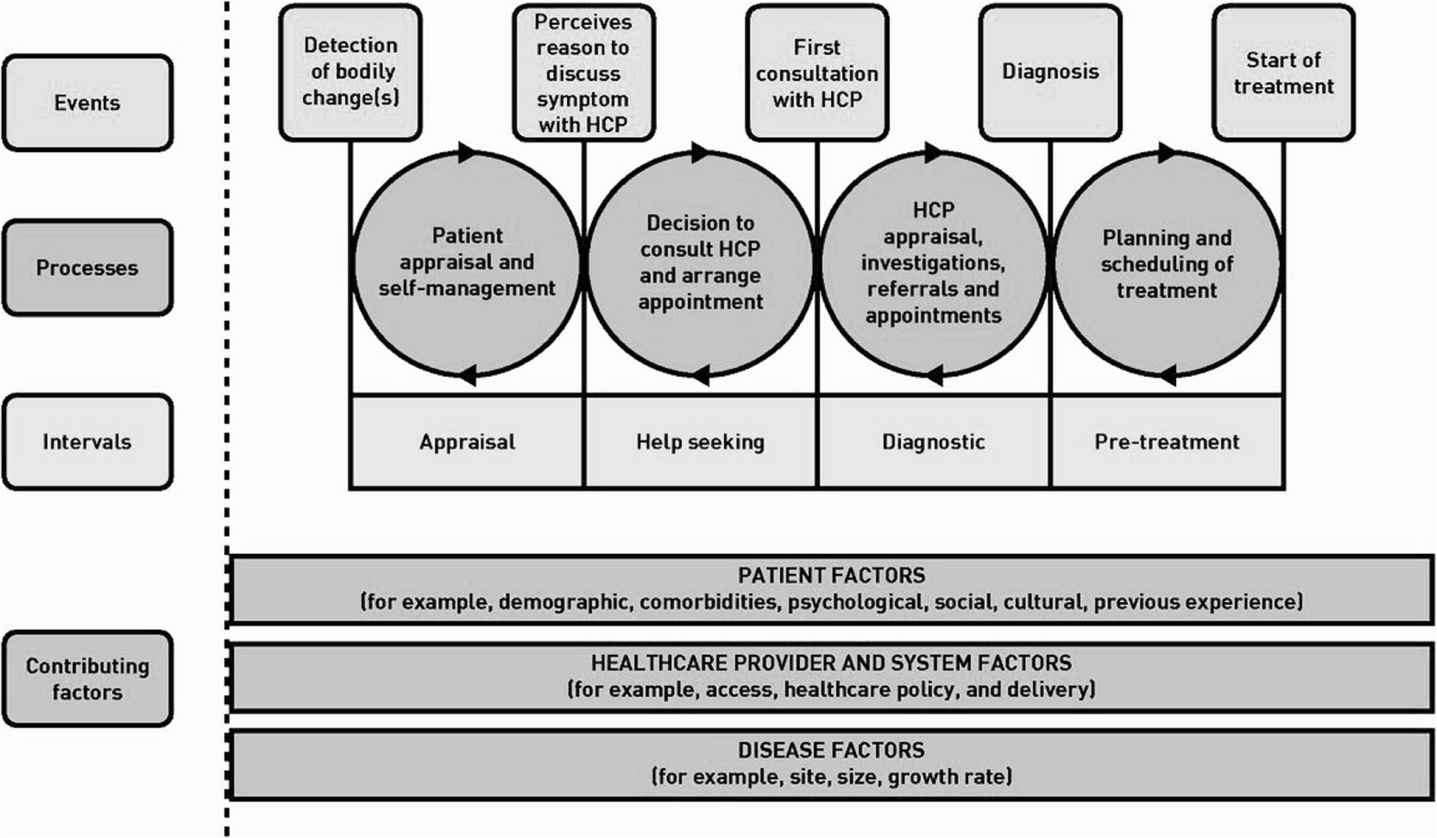
Model of pathways to treatment. Produced with permission from Walter FM [7]

Each interview was coded twice. Initially, they were analysed deductively applying Walter et al.’s model of pathways to treatment (Fig. 2), which provides a theoretical approach for understanding the pathway from detection of bodily or behavioural changes to start of treatment [7, 26]. According to the model, the pathway can be divided into four intervals: 1) Patient (parent) appraisal and self-management (appraisal interval); 2) Decision to consult a health care provider (HCP) and arranging appointment (help-seeking interval); 3) HCP appraisal, investigations, referrals and appointments (diagnostic interval); 4) Planning and scheduling of treatment (pre-treatment interval). We had the appraisal, help-seeking and diagnostic intervals as our focus. Each interval encompasses components specifying the processes and contributing factors related to patient, HCP (e.g. GP) and disease.

Subsequently, interviews were analysed by an inductive-deductive approach, where new topics were added iteratively as they emerged during the coding of interviews. In this way we ensured that any experience which could contribute to a more thorough understanding of the pathway to diagnosis were considered. The researchers discussed any differences in interpretation, until consensus was reached. A summary of each parents’ accounts including descriptions of explanatory factors and mechanisms which influenced their pathway and a time span estimate was made as part of the analysis. Findings from the summaries were merged into themes of factors influencing the pathway to diagnosis. Knowledge about the factors and insights into mechanisms were used in the development of a new explanatory model of the pathway to diagnosis ‘diagnostic triage’ (Fig. 1). Thus, the new model has its base in Walter et al.’s model, adding a new layer of understanding of mechanisms taking place in the appraisal, help-seeking and diagnostic intervals. We believe saturation in the analysis was achieved since no new themes emerged in the subsequent interviews used to validate the diagnostic triage model.

The model was based on the parents’ experiences and validated by member checking with the additional six interviews. Member checking involves the returning of analyses to participants (validating person) for confirmation of accuracy. No revisions on the model were made after the member checking.

## Results

The findings of this study reflect parents’ self-reported experiences of the pathway leading from noticing changes in their child to diagnosis. The parents’ descriptions of their experiences prior to diagnosis provided insights that aided development of the diagnostic triage model, which was subsequently validated. The timespan from first bodily or behavioral changes to diagnosis varied remarkably and ranged from 1 week to 3 years, shortest time span for children with haematological cancer and longest timespan for children with brain tumours (Table 1).

The themes are presented in categories concerning parent’s experiences of everyday life, primary care (GP/Emergency Department) and secondary care (specialised health care in hospital setting). In five themes, we introduce multiple factors that influence appraisal, help-seeking, and obtaining a diagnosis as they are experienced by parents. Finally, we introduce the diagnostic triage model.

### Factors related to everyday life

#### Theme 1: the nature of bodily or behavioural changes

Parents observed various bodily or behavioural changes in their children, mostly described as ‘changes’ and ‘symptoms’. Some changes were of a more general nature, and some more specific, depending on cancer type. Initially, changes were often appraised as non-severe and unspecific or were perceived as common illnesses such as the flu. At this point, parents did not worry particularly leading to a “wait and see” approach instead of seeking help from their GP. A mom explained:

*“He was often extremely sick that winter. But I wasn’t really worried because I thought that kids just get sick all the time when they’re little.” (P12, haematological cancer, Mother).*

For some parents it was difficult to pinpoint when the first change(s) started as they were not sure whether a specific change was related to the initiation of the cancer disease. They used their former experiences as a reference to find logical explanations for their children’s illnesses within the context of their everyday life. Therefore, bodily and behavioural changes were at times normalised and related to age and daily life events, for example growing pains, minor sports injuries or being a teenager*:*

*“We joked about it [lethargy] as being pre-teen behaviour.” (P41, haematological cancer, Father)*.

In some cases, parents even stopped recognising the changes which could prolong the timespan before consulting their GP. Some changes were self-managed e.g. by giving massage or exemption of school sports as families made adjustments within their everyday routines and lives.

However, turns for the worse – persistence, worsening or accumulation of symptoms – affected their daily appraisals and were triggers for help-seeking. Changes presenting as acute-like bleedings often escalated the situation in the home, prompting parents to seek help immediately at an Emergency Department. When not acute, help-seeking was predominantly via the family GP.

In these ways, parents’ appraisal of bodily or behavioural changes in their children contributed to either an escalation of the situation prompting help-seeking or a de-escalation which could prolong the TTD. De-escalation could be due to either misinterpretations or normalization of changes.

#### Theme 2: parental knowledge and intuition

Appraisal was also affected by parents’ intuition and instinctive knowledge about their child. Some parents described how they could “sense” something was wrong. This sense could be indistinct:

*“I can see that she doesn’t feel well, and I can’t say exactly why, but there’s something going on that isn’t the way it’s supposed to be …*” *(P6, solid tumour, Mother).*

They characterised their children as “*sad and moody*”, “*beside himself*” and “… *more vulnerable than usual”.* In general, this parental intuition was a trigger for increased attention and played a significant role in the process of appraisal and in decisions about help-seeking.

In some cases, parents did not agree about their child’s wellbeing. Mothers described how they had repeated discussions with the fathers when they were certain something was wrong, while the father disagreed. Disagreements related to the severity of physical changes, a feeling or a sense. Differing parental appraisals then initiated a negotiation of urgency within the home and discussions about whether a GP should be consulted. In one case, a mother decided to take her daughter to the GP without telling the father. Nevertheless, differences in parents’ appraisal was not reported as a major barrier for help-seeking.

#### Theme 3: social relations

The social context played a significant role in terms of assigning levels of urgency, appraisal and escalation. Family and friends gave parents advice or shared their observations and/or experiences. This would often confirm parent’s concerns and encouraged them to take action e.g. by paying more attention to what had changed and/or by seeking help:

*“Some girlfriends observed that he fell a lot. And I said that I also thought that was the case, but I wasn’t sure if it was just me …” (P45, brain tumour, Mother).*

Knowing people with a medical background could be an advantage which families benefited from. They offered professional and tangible advices which empowered parents act e.g. by changing GPs, contacting a hospital directly or calling the same GP numerous times until the GP would become annoyed enough to make a referral. Parents also experienced how peripheral social relations gave advice when they heard about the child’s symptoms, some of whom possessed relevant experience or medical knowledge:

“*I was talking to a neighbour … and mentioned that she had a slipped disc. She thought that sounded strange, but that her husband knew a lot about it because he was a neurosurgeon. He clearly recommended that she undergo an MR scan and get into the system to have someone look at her.” (P13, solid tumour, Father).*

This neighbourly talk encouraged the family to seek help immediately. Likewise, adult acquaintances such as teachers would also share their observations. A mother described how a teacher had noticed their child was overcome by fatigue in school to an extent they had not recognized at home.

Also, interactions on social media influenced parents’ appraisals and decisions about next steps:

“*I’m part of a Facebook group, where I upload videos and stuff and everyone in there was, like, you need to find someone who can help you because something’s wrong. That’s when I called the hospital …” (P43, brain tumour, Mother).*

In this case, a mom used social media to join a community with parents of children with a severe chronic disease such as the one her son was diagnosed with. She was convinced it was a wrong diagnosis and the feedback from the parents confirmed her suspicions and triggered action.

Parental comments that fall under the theme of social relations clearly demonstrate that the advice of others could turn out to be very important in escalating a situation, prompting help-seeking. Advice was considered and influenced decisions no matter if it came from family members or peripheral acquaintances.

### Factors related to primary care

Parents distinguished between their interactions with their GP and interactions with specialists. The former primarily concerned waiting time related to getting a diagnosis or a referral to specialists. The pathway to diagnosis often involved numerous consultations with GPs as their first point of contact but also opticians, chiropractors, physiotherapists, etc. were sometimes consulted before they obtained a diagnosis.

#### Theme 4: the general practitioner-child-parent interaction

GPs have the power to decide, based on his/her appraisals, whether a case should be escalated with a referral or a positive test moving a child closer to a possible diagnosis or de-escalated, if suspicions are not confirmed. In some cases, the changes and the child’s story were sufficient for a GP to refer or test after one or a few consultations. In other cases, families had multiple visits without any experience of progression. In a few cases frequent visits never triggered a test or referral.

Parents recounted how consultations generated many different experiences and feelings, and they recalled feeling relieved if their child was referred to specialists right away. Initially, parents often felt confident after consultations with their GP, having received plausible explanations of their child’s changes. They felt reassured that nothing serious was wrong and initially decided to “wait and see” if the symptoms would disappear. Diagnosing cancer was challenging for the GPs which initially was acknowledged by many parents. They described how they felt their GP applied a process of ‘elimination’ in the diagnostic process.

The advice of GPs was meaningful and played a significant role in parents’ appraisals in their everyday lives. When a GP confirmed or interpreted the changes as normal or non-severe, parents felt reassured and their worries decreased. They returned to a process of re-appraisal in their everyday life before consulting a GP again. This could continue in a loop moving forwards and backwards between home and GP. Some parents postponed consultations because they were afraid to “*look like a fool*” when returning multiple times to their GP.

But the persistence or worsening of symptoms increased their concern. As time went by with an increasing number of consultations without any experience of progress, parents felt disappointed, frustrated and rejected by their GP. One source of frustration was disagreements with their GP. But also, an experience of insufficient involvement, a feeling of not being listened to and having their knowledge and experiences set aside. Although convinced something was wrong, parents described how being rejected by their GP caused them to doubt their own observations and appraisals:

*“They [the GPs] questioned the validity of my observations as a mother and that made me insanely frustrated and angry. I left my GP so many times doubting my own observations.” (P46, brain tumour, Mother).*

Although disagreements occurred, which could prolong the TTD, decisions could be negotiated which some of the parents took advantage of. They used various strategies in their interactions with the HCS, with the aim of speeding up the process increasingly demanding tests, diagnosis and effective treatment:

*“I had to tell my husband that it was his turn now; he can look quite scary. Now you have to be one of those parents who is supremely annoying, and we have to put our foot down to make them take that blood test.” (P1, haematological cancer, Mother).*

A father used a similar strategy by refusing to leave the room before the GP had taken a blood sample. He escalated the situation and got the test.

Another strategy was used by parents who decided to go their own way in the HCS when the standard care pathways did not meet their needs. This could e.g. be by talking to different GPs or other types of HCPs to get second opinions or navigating around their GP by going directly to a hospital to find out how they could get an appointment as quickly as possible. They benefited from their ability to act strategically in their interactions with the HCS.

In some cases, a good relation to the GP also appeared to influence the pathway. Some parents felt their relation to the GP could speed up the process. A couple of parents described how they insisted to talk to one specific GP. However, in some cases it was consultations with different GPs which were believed to trigger a referral to specialists.

When parents and GPs interact, a negotiation about urgency and action takes places. It influences the triage that continuously takes place in GP consultations which can lead to either de-escalation (families return to re-appraisal in everyday life), which postpones the time to diagnosis, or to escalation, with further examinations and/or referral to specialists where the next level of specialist triage takes place.

### Factors related to secondary care

#### Theme 5: the specialist-child-parent interaction

At the specialist level, waiting time was related to getting a final diagnosis and starting treatment. When children made it to the specialist level, the situation often escalated quickly with targeted tests to identify the precise cancer type and treatment often initiated within days or even hours. Finally, being in the hands of specialists made the families feel safe with an experience of momentary relief when they obtained the diagnosis although followed by feelings of shock. Relief was especially clear for those families who had lived in uncertainty for months. The clinical specialists who were familiar with most of the cancer types were described with a high degree of respect and revere. Still, some parents insisted on talking to a specific specialist.

Even specialists could be challenged, and two couples experienced their children receiving shifting diagnoses from benign to malign. This illustrates how appraisals of symptoms and the level of urgency and triage can also be difficult at the specialist level. At the time of the interview, some children still only had a temporary diagnosis, however, highlighting the complexities of obtaining a specific diagnosis for some in cancer care.

### The pathway to diagnosis explained by diagnostic triage

In often-busy everyday lives filled with jobs, hobbies, family obligations etc. parents continuously appraise the well-being of their children. In cases of concern about their child, parents can choose to escalate the situation and act based on their appraisal. The escalations that ultimately prompt help-seeking were shown to be influenced by multiple factors related to themes of the nature of the bodily and behavioural changes, parental intuition and social relations. Likewise, situations were also de-escalated and help-seeking postponed possibly prolonging the TTD. We designate this dynamic continual process of appraisal/re-appraisal and escalation/de-escalation ‘everyday triage’.

Diagnostic triage is a sequential model (Fig. 1) covering three different forms of triage that take place from the moment a parent/child notices bodily or behavioural changes and continues until a diagnosis has been received, each step encompassing different levels of power and knowledge. Triage involves assigning levels of urgency, in our case, not so much in connection with treatment but rather in diagnostic pathways. Focusing on diagnostic triage sets the everyday life of families at the centre of the analysis. Parents make decisions daily on behalf of their child through a form of everyday triage. They are vigilant about the wellbeing of their child, making appraisals and judgements about any change(s) that might indicate their child should stay home from day care or school, or that an appointment with an GP or another HCP should be made. When appointments are made, the types of assessment that characterise diagnostic triage move into the HCS, where we distinguish between general triage (GP/Emergency Department) in primary care and specialist triage (Hospital) in secondary care.

Clearly, multiple factors affect the pathway to diagnosis and, consequently, what we term ‘diagnostic triage’. In the ways we have shown in our analysis, the processes of appraisal/re-appraisal, help-seeking, negotiation and escalation/de-escalation were iterated continuously until a diagnosis was obtained.

## Discussion

This study explored and analysed the time between noticing bodily or behavioural changes and diagnosis, from the perspective of parents of children with cancer. It proposes an explanatory model called diagnostic triage (Fig. 1) which explains everyday mechanisms that shape the pathway and TTD from the detection of bodily or behavioural change(s) to diagnosis. Diagnostic triage consists of dynamic movements between different ways of assigning urgency regulated by appraisal/re-appraisal, help-seeking, negotiation and escalation/de-escalation (Fig. 1). While de-escalation can prolong the TTD in the case of cancer, escalation will more rapidly move the child closer to a possible diagnosis. As we have shown, parental appraisals can lead to escalation through increased concern and prompt help-seeking (everyday triage). After contacting the HCS, predominantly their GP, he or she can either further escalate a case (e.g. by taking a blood sample) or de-escalate, if further clinical actions are deemed unnecessary, with families then returning to the daily re-appraisals that characterise everyday triage.

Previous studies have shown that delay occurs at different stages during diagnostic pathways due to a variety of factors [8–11, 30–32]. This is in line with our findings as the pathway to diagnosis was shaped by factors related to disease, patient/parents and the HCS. Often, initial changes did not lead to concern and changes were at times normalized and adjusted to in everyday life. However, parental appraisal of their child’s wellbeing could change, possibly due to a change or accumulation of symptoms and/or the intuition that something was wrong which prompted help-seeking. The presence of many different symptoms complicating the diagnostic process of a childhood cancer diagnosis have also been documented in the literature [33, 34]. Interpretation of bodily or behavioral changes and health behavior is embedded within a social and cultural context [27, 35–37]. Family members and acquaintances influenced the pathways by commenting on the appearance and behavior of children, giving advice and sharing knowledge, which in turn affected parents’ appraisals and decisions about help-seeking. The significant role of social relations during the diagnostic process and cancer care is in line with findings in the literature [31, 38, 39].

After seeking help, the interaction and communication of parents and GPs during the process of obtaining a diagnosis is central. Similar to our findings, studies have shown disagreements between parents to children with cancer and GPs concerning seriousness of symptoms and the need for testing [8, 9, 11, 40]. Other studies suggest that parents might not use red flag words or experience red flag symptoms [8, 33]. Both things could lead to de-escalation even in the case of cancer. Moreover, parents have experienced GPs as failing to address or include their insights, causing them to feel that the GPs discounted their intimate knowledge of their child [9, 11]. This was also in line with experiences from our study with parents not feeling sufficiently involved.

### Diagnostic triage: a revised model of the pathway to diagnosis

To our knowledge there are no existing explanatory models of the everyday mechanisms shaping the pathway to diagnosis based on the perspectives of parents of children with cancer. A number of models have been developed in an attempt to describe events and processes along the pathway to a diagnosis [7, 36, 41–44]. Existing models often divide the period prior diagnosis into intervals with specific starting and ending points.

Overall, the model of pathways to treatment [7] provided a useful coding frame for the analysis supplemented by the inductive-deductive coding. However, the starting point “onset of symptoms” was rigid and not applicable in the analysis of the parent’s descriptions. In many cases the parents were not sure whether a symptom or change was related to the cancer. This was also pointed out in a previous study on patient’s experiences [45]. The model of pathways to treatment (Fig. 2) illustrates pathways with double arrows to represent the complexity of pathways as they cannot be described as linear. Diagnostic triage revises the model and demonstrates the non-linearity and the complexity of the factors and mechanisms shaping the pathway to diagnosis and thereby contributes to explain variation in the pathways. We elucidate how various factors including context and different levels of power and knowledge are key players influencing the pathway to diagnosis. We have presented the various factors shaping the pathway to diagnosis as consistent parts of the diagnostic triage model as each factor impacts the levels of urgency assigned by parents or HCPs to observed changes and thus the TTD.

It is on this basis that we propose diagnostic triage as an explanatory model to understand the mechanisms shaping the pathway from detection of change(s) in a child by parents to diagnosis. The model captures the dynamic movements between forms of diagnostic triage – which is to say assignments of urgency in terms of seeking an explanation for affective, bodily or behavioural changes – in everyday life and in the HCS, both of which are regulated by various factors affecting appraisal, help-seeking, negotiation and (de-)escalation. These recurrent activities illustrate a pathway that should be perceived as a sequential model based on negotiations between children, parents and GPs and/or other HCPs.

### Diagnostic triage and inequality

Delay has been raised as a possible explanation for social inequality in utilization of primary care [14] and survival among children with cancer, also within the Danish welfare state [46]. We are aware, the study design does not allow any conclusions about the influence of socioeconomic position [47] on events or outcomes along the cancer trajectory. Nonetheless, we believe the model is relevant for investigating social inequality as it enables separate exploration of triage and movements (i.e. between everyday, general and specialist triage) to better understand how socioeconomic position and contexts affects the various processes, escalations and, in the end, TTD.

Our study showed how variations in parents’ interactions with the HCS may reflect differences in the social and cultural capital of each family. This is based on the theory of interaction as a site for capital generation and exchange [48, 49] which enables some parents to successfully negotiate with GPs for testing/referral or to draw on other resources thereby affecting diagnostic triage. Studies have described how health values and norms, knowledge and operational skills are key elements of health-relevant cultural capital and highly relevant for explaining inequality in health [50, 51]. We suggest, parents who acted strategically took advantage of their social and cultural capital on their pathway to diagnosis by interacting and pushing forward, thereby possibly reducing TTD. We recommend further studies of how social and cultural capital can influence diagnostic triage processes.

### Strengths and limitations

#### Strengths

We examined the unique perspectives of parents. With the child at the centre, parents are the only people who possess intimate knowledge about the obstacles, barriers and uncertainties on the road to a diagnosis. The data consisted of self-reported experiences in the form of retrospective accounts of pathways to diagnosis. Parents, however, have been shown to be reasonably accurate in recalling their diagnostic experiences [25]. We included parents of children with various types of cancer. Although the TTD varies by cancer type [13] previous studies have shown similarities between experiences of delay among different types of cancer [52].

#### Limitations

Parents in the last six interviews were selected by experienced specialists who deemed the children had had a noticeably long and/or frustrating pathway to diagnosis. The specialist compared the children’s trajectories and TTD with more typical trajectories and TTD for children with similar diagnoses. This judgement was based on the individual specialists without further definitions, and thereby involves a degree of subjectivity which introduces possible bias in the sample. This concern of bias is documented in the literature showing a lack of agreement between clinicians regarding what is an appropriate interval [27]. Member checking was done to ensure that “the participants own meanings and perspectives are represented and not curtailed by the researchers’ own agenda and knowledge” [53]. However, member checking has been criticised for not recognising that researchers and participants may have different goals [54].

Some parents were interviewed within a few days after their child’s diagnosis and some were interviewed months later. Furthermore, some families had only received a temporary diagnosis at the time of the interview. This could all impact what the parents recall.

## Conclusions

In this study, we have proposed an explanatory model called ‘diagnostic triage’ (Fig. 1) which explains everyday mechanisms that shape the pathway and TTD from the detection of bodily or behavioural change(s) to diagnosis. The model identifies dynamic movement between parental triage in everyday life and professional triage in a healthcare system, both affecting appraisal and case escalation according to: 1) the nature of bodily and behavioural changes, 2) parental intuition, 3) social relations, 4) general practitioner-child-parent interaction, and 5) specialist-child-parent interaction. The model can be viewed as generic and transferable to other disease trajectories. What is more, our diagnostic triage model has been developed to support the clinical diagnostic process, and also as a novel theoretical framework for future research on diagnostic pathways.

Early diagnosis of childhood cancer is required to avoid unnecessary progress of disease. GPs are faced with a dilemma as they are also charged with avoiding overtreatment, over-testing or referring too early, as many children might be subjected to invasive or traumatic tests for no reason. They need to balance clinical issues by escalating the “right ones” by testing and/or referring and de-escalating the “others” according to guidelines and clinical sense in order to ensure a secure and efficient HCS, not least in a welfare state where efficiency savings are common. Parents and GPs may approach consultations in different ways. Parents seek help when they notice affective, bodily or behavioral change(s) in their child interfering with his/her well-being or when people from their surroundings advise them to do so. Conversely, a GP’s approach is to diagnose based on symptoms and objective information derived from reports from parents, examinations and test results. Changes and illness are experienced by patients in a social and cultural context which is important to the diagnostic process. Therefore, we need to ensure that factors influencing everyday forms of triage are considered in dialogues between patients/parents and GPs. The diagnostic triage model has the potential to support GPs in their communication with parents that moves beyond descriptions of physical symptoms, e.g. by paying attention to whether social relations have commented on changes in a child. It is relevant for the development of teaching materials and tools to support the GP, that ultimately could ensure more timely testing, referral and diagnosis. This paper is a contribution to discussions about how evidence based medicine needs to incorporate sociocultural factors, such as family influences and patient values and preferences [55].

## Supplementary information

**Additional file 1.** Semi-structured interview guide and questions for member checking.

## Data Availability

The datasets used and/or analysed during the current study are available from the corresponding author on reasonable request.

## Abbreviations

GP: General practitioner
HCP: Health care provider
HCS: Health care system
TTD: Time to diagnosis
