# Supplementary material for: Parent’s perspectives of the pathway to diagnosis of childhood cancer: a matter of diagnostic triage
Source: BMC Health Serv Res. 2020 Oct 22;20:969. doi: 10.1186/s12913-020-05821-2 (PMC7584100; doi:10.1186/s12913-020-05821-2)
Supplement: Supplementary file 1 — Additional file 1. Semi-structured interview guide and questions for member checking. [file 12913_2020_5821_MOESM1_ESM.pdf]

Interview guide: Parent's perspectives of the pathway to diagnosis

Themes and main questions are further explored by follow-up questions and examples.

Background information

Parent/other, cohabitation status/children, affiliation to work market, education, ethnicity.

- What type of cancer does your child suffer from?

- When was he/she diagnosed?

Introductory question

- Try in your own words to describe how you ended up here? (supported by questions below)

Noticing changes / help-seeking

- How did you experience your child's changes/symptoms before diagnosis? (appraisal/self-management)

- Can you describe your considerations regarding help-seeking? (knowledge, experiences, involvement of others, triggering factors)

Interactions with the health care system

-How did you experience consultations/interactions with the health care system/health care providers? (type of contacts/investigations/referrals, roles, involvement/decision-making, obstacles)

Diagnosis and concluding questions

- Can you describe the time around diagnosis? (investigations, roles, reactions)

- How did you experience the process of obtaining the diagnosis? (challenges/barriers)

- Is there anything else that is important to understand your experiences of the pathway to diagnosis?

STEP 2: Validation of model (member checking)

Validation of the model of diagnostic triage was done by member checking interviews (interview number 27-32). The member checking interviews were divided into two steps:

Step 1) Semi-structured interview supported by interview guide (see page 1).

Step 2) Member checking (questions below).

Interviewer:

- Introduce the model of diagnostic triage.
- Explain the background and the purpose of introducing the model as the final part of this interview.
- Explain what the parents can contribute with in terms of validation or further development of the model based on their experiences.
- We are interested in their perspectives of whether the model reflects their experiences. We would like the parents to explain how the model does – or does not - reflect their experiences.

Questions for the parents (member checking):

- Please describe if you think the model is an accurate way of framing/illustrating your child's pathway to diagnosis? Please elaborate.
- Can you try to describe where you see similarities or differences in the model that do or do not match your experiences of the pathway to diagnosis?
- Do you find anything missing/inaccurate in the model in relation to your experiences?
- Do you recognize the way of thinking the pathway to diagnosis?
- Do you have any other comments to the model we should consider?
